# Anthocyanins Found in Pinot Noir Waste Induce Target Genes Related to the Nrf2 Signalling in Endothelial Cells

**DOI:** 10.3390/antiox11071239

**Published:** 2022-06-24

**Authors:** Jesús Herrera-Bravo, Jorge F. Beltrán, Nolberto Huard, Kathleen Saavedra, Nicolás Saavedra, Marysol Alvear, Fernando Lanas, Luis A. Salazar

**Affiliations:** 1 Center of Molecular Biology and Pharmacogenetics, Scientific and Technological Bioresource Nucleus, Universidad de La Frontera, Temuco 4811230, Chile; jehebra2@gmail.com (J.H.-B.); nolberto.huard@ufrontera.cl (N.H.); kathleen.saavedra@ufrontera.cl (K.S.); nicolas.saavedra@ufrontera.cl (N.S.); fernando.lanas@ufrontera.cl (F.L.); 2 Departamento de Ciencias Básicas, Facultad de Ciencias, Universidad Santo Tomas, Temuco 4700000, Chile; 3 Department of Chemical Engineering, Faculty of Engineering and Sciences, Universidad de La Frontera, Temuco 4811230, Chile; beltran.lissabet.jf@gmail.com; 4 Department of Chemical Sciences and Natural Resources, Faculty of Engineering and Sciences, Universidad de La Frontera, Temuco 4811230, Chile; marysol.alvear@ufrontera.cl; 5 Department of Internal Medicine, Faculty of Medicine, Universidad de La Frontera, Temuco 4700000, Chile

**Keywords:** grape pomace, oxidative stress, bioactive compounds, protective effect, agro-industrial waste, wine industry, anthocyanins, HO-1, NQO1

## Abstract

Grape pomace is a source of anthocyanins, which can prevent cardiovascular diseases due to their antioxidant properties. Anthocyanin activity is associated with the ability to regulate oxidative stress through the transcription factor Nrf2. Thus, the present study aimed to evaluate if the anthocyanins found in Pinot noir pomace extract can affect the target genes related to the Nrf2 signalling pathway in endothelial cells. Our results highlight that the predominant anthocyanin in the Pinot noir pomace extract was malvidin-3-glucoside (3.7 ± 2.7 Eq. Malv-3-glu/kg). Molecular docking indicated that cyanidin-3-glucoside (−6.9 kcal/mol), malvidin-3-glucoside (−6.6 kcal/mol) and peonidin-3-glucoside (−6.6 kcal/mol) showed the highest affinities for the binding sites of the BTB domains in Keap1, suggesting that these components may modify the interaction of this protein with Nrf2. In addition, when HUVEC cells were exposed to different concentrations of Pinot noir pomace extract (100 µg/mL, 200 µg/mL, and 400 µg/mL), no changes in Nrf2 gene expression were observed. However, the gene expression of HO-1 and NQO1, which are in the signalling pathway of this transcription factor, increased according the concentrations of the extract (*p* = 0.0004 and *p* = 0.0084, respectively). In summary, our results show that anthocyanins play a very important role in Nrf2 activation and release, while at the same time not promoting its transcription. These preliminary results strongly suggest that the Pinot noir pomace extract can serve as a potent bioactive component source that protects cells against oxidative stress.

## 1. Introduction

In recent years, there has been a growing concern about environmental pollution linked to the generation of agro-industrial waste. The wine industry generates approximately 8.49 million tons of grape pomace per year worldwide; this residue can be used to obtain compounds with biological activity [1]. The recovery of these compounds could represent an important step in maintaining the balance of the environment [2]. Wine producing regions are mainly in Europe (Italy, Spain, France, Germany and Portugal) and America (USA, Argentina and Chile), but also in Australia and South Africa [3]. Even though Chile has experiences in the use of pomace, converting it, for example, into hydrocarbon, uses explained in Garrido et al. [4], it is necessary to promote innovation for the correct use of the waste produced by the wine industry, and to add value to the wine industry, which is so important to the country.

Grape pomace is a source of anthocyanins, pigments with antioxidant properties that help prevent cardiovascular diseases [1]. These molecules are one of the biggest families of pigments in the plant kingdom. They are responsible for the blue, purple, red and orange colours present in fruits and vegetables. Chemically, anthocyanins are anthocyanidin glycosides that belong to the flavonoid family, consisting of two aromatic rings (A, B) linked with a heterocycle (C) [5]. To date, more than 500 different anthocyanins that differ not only for the glycosylation pattern of the scaffold, but also for the presence and position of aliphatic or aromatic carboxylates are reported [6]. In this way, anthocyanin compounds are effectively used in analytical chemistry for variety and species recognition by chemical fingerprinting techniques [7,8]. Cyanidin (Cy), Delphinidin (Dp), Pelargonidin (Pg), three among the non-methylated anthocyanidins, are the most common in nature [6], the same ones that are seen on the skin of the grape [9]. The anthocyanins most distributed in plants are those originated by Cy, Dp, and Pg (indeed they are present in 80% of the leaves, 69% of the fruits, and 50% of the colored flowers, meanwhile those formed by Pt, Mv, and Pn are limitedly distributed) [6,10,11]. Concerning their chemical structure, anthocyanins are very unstable, easily oxidized, and sensitive to many factors, such as pH, which affects their stability and colour, temperature, and ultraviolet radiation [12]. However, as a consequence of glycolation, anthocyanins acquire greater solubility and stability than those without glycosylation [6,13,14].

Today, the use of anthocyanins is important in the treatment of cardiovascular diseases, and their pharmacological effect attributed to their chemical structure, such as the antioxidant potential of anthocyanins depends on the number and position of the hydroxyl groups, the degree of glycosylation and the presence of unpaired electrons in their rings [15]. In this context, malvidin-3-glucoside has been studied as a good antioxidant [16], since it can be expected that the consumption of fruit or vegetables that possess this anthocyanin could exert an anti-atherogenic effect via regulation of oxidative stress.

A great number of investigations suggest that nuclear factor-erythroid factor 2-related factor 2 (Nrf2) is a key molecule to inflammation and oxidative stress response. Nrf2 is a member of the Cap’n’collar family of transcription factors, containing 605 amino acids and is divided into seven well-conserved domains [17]. Of this structure, the N-terminal domain influences the stability and ubiquitination of Nrf2 by its negative regulator Kelch-like ECH-associated protein 1 (Keap1), while the Neh5 domain is responsible for the cytoplasmic localization of Nrf2 [18]. The Neh1 domain has an important role in regulating binding to the DNA molecule and is involved in nuclear translocation. The Neh2 domain has a fundamental role in binding to the Keap1 protein through the DLG and ETGE degrons [19]. Keap1, the main regulator of Nrf2, consists of 624 amino acids and is classified as a protein rich in cysteines, since it has 27 residues of this amino acid [18]. Keap1 has five domains, of which the BTB domain is relevant since it has been studied to play an important role in the activation of Nrf2 in response to oxidative stress [20]. The Kelch domain allows binding to Nrf2 through the Neh2 domain [21].

The activation mechanism of Nrf2 has been well documented [14,16,17]. Under normal conditions, Nrf2 has a short life of approximately 20 min, after which it is degraded at the proteosomal level, remaining in low concentrations. The Keap1-Nrf2 regulation pathway is based on the hypothesis that when there is a deregulation of the homeostatic balance after ROS presence or the activation of Nrf2 through inducers, Keap1 loses its structure interfering in the interactions between the Kelch domain and the DLG and ETGE degrons [22]. Consequently, the process targeting the ubiquitination and degradation of Nrf2 failed, causing the accumulation of Nrf2 in the cytoplasm, translocating to the nucleus, allowing the expression of genes that are in the regulation cascade of this pathway. Under conditions of oxidative stress, oxidation of Cys151 residues of Keap1 causes conformational changes in Keap1 allowing the release and activation of Nrf2 [23].

The activation of Nrf2 has been evidenced in response to the in vitro protective effect of extracts generated from various natural products [24,25,26]. Genes linked to the regulation of oxidative stress express once Nrf2 translocate into the nucleus [27]. Among the widely studied genes are those that allow the translation of Heme oxygenase 1 (HO-1) and NAD(P)H Quinone Dehydrogenase 1 proteins (NQO1). HO-1 is an enzyme that catabolizes the Heme group, generating equimolar amounts of Biliverdin, ferrous iron, and carbon monoxide [28]. In a general setting, HO-1 modulates the expression of adhesion molecules associated with endothelial cell dysfunction. This effect has been associated with biliverdin production [29]. Likewise, carbon monoxide (CO) has shown cytoprotective effects, including anti-inflammatory and antioxidant effects. A study conducted by Cheng determined that CO released by carbon monoxide releasing molecule-2 (CORM-2) inhibits inflammatory responses induced by particulate matter in human oral keratinocytes (HOK) [28]. At low concentrations, CO inhibits vasoconstrictor enzymes such as Endothelin-1, as well as TNFα and Interleukin 1 (pro-inflammatory interleukin) and increases Interleukin 10 (anti-inflammatory interleukin) [30]. NQO1 is a highly inducible protein in response to multiple forms of stress, including oxidative stress and many protective mechanisms against oxidative damage [31], so its evaluation in this work presents an opportunity to recognize its action against mechanisms linked to the protection of endothelial cells.

To better understand the pharmacological properties of Pinot noir pomace extract, we investigated the effect of anthocyanins found in the extract on the gene expression of HO-1 and NQO1, targets related to the Nrf2 signalling, in endothelial cells.

## 2. Materials and Methods

### 2.1. Sampling

The samples were obtained from the El Capricho sector in Galvarino commune, Valle del Cautín, La Araucanía Region, Chile.

#### 2.1.1. Bunch

The grape samples were collected in a zig-zag pattern inside the vineyard. Cluster samples were taken from the east and west sectors of the trellis without considering the edges of the vineyard, which were near the main road. Berries obtained from Pinot noir bunches were used as a control.

#### 2.1.2. Fermented Pomace

The fermented pomace was sampled once the pressing was finished (pressing time = 1 h). The pomace with seed stems and finished wine go through a vertical press until reaching 1 bar of pressure manually. Subsequently, the pomace cake is disassembled by extracting the sample from the middle part and ends of the hat. The sample was stored at −20 °C until analysis. 

### 2.2. Sample Preparation

Pinot pomace was dried at 40 °C in a Zhicheng oven model ZFD-A5090 (Zhicheng, Shanghai, China) for 72 h. The pomace was separated, leaving branches outside the sample to be analyzed, keeping the skin and the seed inside. After drying, 10 g were put into a 200 mL Erlenmeyer flask, and 50 mL of ethanol with 1% formic acid was added. The extract was sonicated for 20 min using an Elmasonic E60H (Elmasonic, Singen, Germany) and then transferred to a Quimis thermoregulated bath for extraction at 30 °C for 30 min. The extract was filtered, vacuum dried in a BUCHI R-210 rotary evaporator (BUCHI, Essen, Germany) and then lyophilized to be dissolved in methanol for further analysis. The extraction process was completed in triplicate. The antioxidant power and the number of total polyphenols of the pomace were compared with the Pinot noir grape rescued before the vinification process.

### 2.3. Chemical Characterization of the Extract

#### 2.3.1. Total Monomeric Anthocyanin Content

Total anthocyanin quantification was performed using the differential pH method as published by Lee, Durst, and Wrolstad (2005) [32] and following the modifications made by Melo et al. (2015) [33]. This spectrophotometric method is based on the structural transformation of anthocyanins with a change in pH (pH 1.0 coloured and pH 4.5 uncoloured). The extract was diluted in a solution of Potassium Chloride (0.025 M pH 1.0) and Sodium Acetate Buffer (0.4 M pH 4.5) in a 4:1 ratio (buffer: sample [20,000 µg/mL]). The solution was incubated for 20 min in the dark and the absorbance was performed at 520 and 700 nm against the buffer and acid solution as blanks. The absorbance was converted to total mg of Malvidin 3-glucoside (molecular weight 494 g/mol; ε = 36,400 L cm^−1^ mg^−1^) per gram of dry mass (Equation (1)). The samples were analyzed in biological triplicates with approximately 15 technical replicates.

(1) $$Total\;anthocyanins\left(mg/L\right)=\frac{A\times PM\times 1000}{\varepsilon \times 1}\times FD$$

where: A (absorbance) = (A_520nm_ − Abs_700nm_)_pH1_ − (A_520nm_ − A_700nm_)_pH4.5_; PM = molecular weight of Malvidin-3-glucoside: 494 g/mol; DF = Dilution factor; ε = 36,400 L cm^−1^ mg^−1^, molar extinction coefficient for Malvidin-3-glucoside.

#### 2.3.2. Anthocyanin Analytical Methodology

The quantitative analysis of the extract was carried out by HPLC using an Agilent model 1200 equipment and a LiChrospher 250-4 RP-18 (5 μm) in gradient mode at room temperature and under the same conditions described in Herrera et al. (2021) [34]. The injection was 150 μL with an adequate flow to the gradient and a maximum time of 45 min. A scan was carried out, and it was measured at 520 nm, the wavelength being described for anthocyanins. The mobile phase was composed of: Solvent A: Formic Acid 10% in water; Solvent B: Acetonitrile. The running conditions were set as follows: 0 min 96% A, 4% B, 7.9 min 85% A, 5% B, 8 min 85% A, 15% B, 22.9 min 85% A, 15% B, 23 min 85% A, 15% B, 27 min 80% A, 20% B, 40 min 70% A, 30% B, 43 min 70% A, 30% B, 43.1 min, 96% A, 4% B, 45 min 96% A, 4% B. The identification of malvidin-3-glucoside was carried out by comparison of its spectra and retention time with its standard. The calibration curve of malvidin-3-glucoside was obtained at 520 nm by injection of different volumes of standard solutions. The conditions in which the standard was quantified were the same conditions as for the analyzed sample. Other components for which no standards were available, cyanidin-3-glucoside, delphinidin-3-glucoside, peonidin-3-glucoside and petunidin-3-glucoside, were identified by their spectral parameters.

#### 2.3.3. Oxygen Radical Absorbance Capacity (ORAC)

The antioxidant capacity of Pinot noir pomace extract was evaluated using the ORAC method. Briefly, 10 g of pomace extract was centrifuged for 10 min and then filtered through a Whatman grade 40 membrane. The extract was diluted 1000-fold in 75 mM PBS pH 7.0. Subsequently, 0.046 g of AAPH (2,2-azobis (2-amidino-propane) dihydrochloride) were diluted in 10 mL of 75 mM PBS (pH 7.0) reaching a final concentration of 160 mM. The fluorescein (1.2 mM) working concentration was prepared with 75 mM PBS pH 7.0 and stored at 4 °C in the dark. Trolox stock solution was prepared by dissolving 25 mg in 50 mL of 75 mM PBS pH 7.0 (2 mM). The calibration curve was made with the following concentrations of Trolox: 100 µM, 50 µM, 25 µM and 12.5 µM, each of them diluted in PBS. Fluorescence was measured in a Perkin Elmer 2030 VICTOR X2 spectrofluorometer (PerkinElmer, Turku, Finland) and immediately measured after adding AAPH until the intensity was less than 5% of the initial reading. ORAC values are expressed as µmol Equivalents of Trolox/100 g of dry mass, calculated using the following formula:

(2) $$ORAC=\frac{\left(AUC_{Sample}-AUC_{Blank}\right)}{\left(AUC_{Trolox}-AUC_{Blank}\right)}\times C_{Trolox}K$$

where: AUC (Area under the curve); C (Trolox standard concentration) and K (Sample dilution factor).

### 2.4. Molecular Docking

In this study, we evaluated the affinity of polyphenols and anthocyanins on the BTB and IVR domain of Kelch-like ECH-associated protein 1. For this purpose, the structure of Kelch-like ECH-associated protein 1 (ID: AF-Q14145-F1) which was determined with the AlphaFold program [35] was downloaded from the AlphaFold Protein Structure Database [36]. This protein was truncated with the PyMOL software (The PyMOL Molecular Graphics System, Version 2.0, San Diego, CA, USA) in order to avoid undesired unions with respect to the BTB and IVR domains under study. In this sense, the resulting truncated fragment of Kelch-like ECH-associated protein 1 is composed of amino acids from 56 to 313 a.a. Subsequently, a blind molecular docking was performed with the Autodock Vina program [37] which is integrated in the PyRx software [38], taking as input the Kelch-like ECH-associated protein 1 fragment as receptor and several polyphenols and anthocyanins as ligands. Prior to molecular docking, all ligands were minimized with the mmff94 force field which is also available on PyRx. The evaluated polyphenols and anthocyanins were downloaded from the PUBCHEM database in SDF format as follows: (Polyphenols) quercetin-3-rutinoside (CID: 5280805), quercetin-3-glucoside (CID: 5280804), quercetin-3-galactoside (CID: 5281643), quercetin (CID: 5280343), protocatechuic acid (CID: 72), procyanidin gallate (CID: 15593124), kaempferol-3-glucoside (CID: 5282102), gallic acid (CID: 370), ferulic acid (CID: 445858), catechin (CID: 9064), and astilbin (CID: 119258). (Anthocyanins) cyanidin 3-glucoside (CID: 441667), delphinidin 3-glucoside (CID: 443650), malvidin-3-glucoside (CID: 443652), peonidin-3-glucoside (CID: 443654), and petunidin 3-monoglucoside (CID: 443651). The parameters used for molecular docking were exhaustiveness = 8, and an interaction box with the following dimensions: center_x = −19.77, center_y = −4.21, center_z = 19.62, size_x = 75.69, size_y = 71.80, and size_z = 54.88, which covers the entire fragment. Finally, all molecular docking performed were analyzed and visualized using the PyMOL software.

### 2.5. Cell Culture

#### 2.5.1. Culture Medium

Human Umbilical Vein Endothelial Cells (HUVEC, Product number 200P-05N) were cultured in endothelial cell growth medium (Sigma Aldrich^®^, Darmstadt, Germany), supplemented with sodium pyruvate [0.11 g/L], sodium bicarbonate [3.7 g/L], penicillin-streptomycin [100 units/mL] and 10% FBS (GIBCO) at 37 °C and 5% CO_2_. The culture medium was renewed every 48 h, and when the cells reached 80% confluence, they were disaggregated and seeded at the appropriate concentration to carry out the cell viability protocols. A part of them were cultivated under the same conditions for the repetition of the viability experiments. Cells were only used up to passage number eight.

#### 2.5.2. Reverse Transcription Followed by Polymerase Chain Reaction (RT-qPCR)

The gene expression of NRF2, NOQ-1 and HO-1 was evaluated using the 2^−ΔΔCt^ method [39]. RPL30 was used as normalizing gene.

##### Total RNA Extraction

The total RNA was extracted using Trizol reagent (Invitrogen, Waltham, MA, USA), following the manufacturer’s instructions. Approximately 75,000 cells were cultured in 12-well plates and then lysed with 380 µL of Trizol reagent, and the homogenized mixture was incubated for 5 min at room temperature to allow dissociation of histone proteins. Next, 76 µL of chloroform was added, and the sample was vigorously mixed for 15 s and incubated at room temperature for 3 min. Subsequently, they were centrifuged at 12,000× *g* for 10 min at 4 °C. The upper aqueous phase was transferred to a tube with 190 µL of isopropyl alcohol, incubated at room temperature for 10 min and centrifuged under the aforementioned conditions, allowing the precipitation of the RNA contained in this phase. The obtained pellet was washed with 380 µL of 75% ethanol and centrifuged at 12,000× *g* for 5 min. The supernatant was discarded, and the precipitate dried for 10 min. Finally, total RNA was resuspended in 40 µL nuclease-free water. Its concentration and purity were determined using a NanoQuant infinite M200PRO TECAN spectrophotometer (Tecan, Grödig, Salzburg, Austria) by its absorbance ratio A260/280.

##### Synthesis of Complementary DNA

Once the concentration of the RNA extracted from the HUVEC cells was known, the necessary volume was calculated so that its final concentration was 100 ng/µL. Subsequently, cDNA synthesis was carried out following the protocol of the High-Capacity RNA-to-cDNA Master Mix Reverse Transcription Kit (Applied Biosystems, Bedford, MA, USA). Briefly, total RNA was dissolved in 2 µL 10× RT Buffer, 0.8 µL 25× dNTP Mix (100 mM), 2 µL 10× RT Random Primers, 1 µL MultiScribe RT and 1 µL RNAse Inhibitor. The mixture was made up to a final volume of 10 µL with nuclease-free H_2_O. Subsequently, samples were incubated for 10 min at 25 °C, followed by 120 min at 37 °C and 5 min at 85 °C in a MultiGene OptiMax thermocycler (Labnet, Edison, NJ, USA). The synthesized cDNA was frozen at −20 °C until use.

##### Relative Quantification by Real-Time PCR

The PCR reaction was carried out using the following conditions: 200 mM of each primer, 10 µL of Fast SYBR Green Master Mix (Thermo Fisher Scientific, Waltham, MA, USA), 1 µL of cDNA and sterile distilled water for a final volume of 20 µL. Subsequently, they were subjected to the following thermocycling scheme in a StepOne Plus equipment (Applied Biosystems, Bedford, MA, USA): initial denaturation at 95 °C for 5 min, followed by 40 compound denaturation cycles at 95 °C for 5 s. Negative controls were included in all series of reactions. The nucleotide sequences of the primers designed through the platform of the National Center for Biotechnology Information (NCBI) (http://www.ncbi.nlm.nih.gov/tools/primer-blast. accessed on 27 October 2021) were used. The primer sequences used are described in Table 1.

### 2.6. Statistical Analysis

The normal distribution of the data was evaluated through the D’Agostino normality test. The ROUT method was used to find outliers. The Mann-Whitney test was used to compare total anthocyanin concentrations and antioxidant power in pomace. One-way ANOVA was used for comparison of gene expression of Nrf2, HO-1 and NQO1. For all trials, differences are considered statistically significant when the *p*-value is <0.05. Statistical analysis was performed with GraphPad Prism software (GraphPad Software, Inc., San Diego, CA, USA) for MAC and PC.

## 3. Results and Discussion

### 3.1. Total Monomeric Anthocyanins and Antioxidant Activity of Pinot Noir Pomace

**Figure 1:**
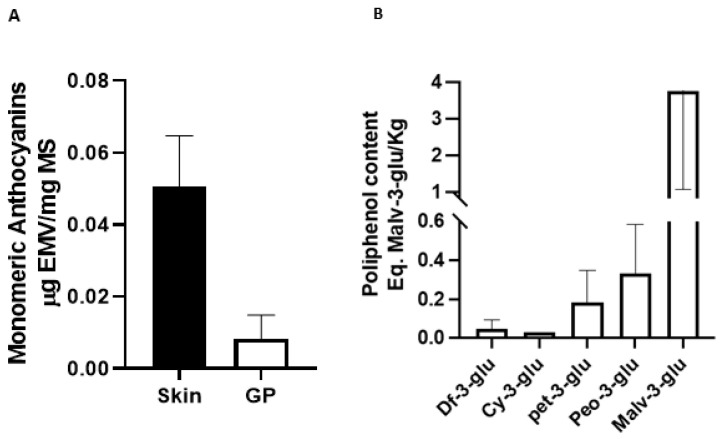
Total anthocyanin content (Eq. Malv-3glu/Kg) of two samples from both HPLC systems (**A**) and the pH differential method (**B**).

Total monomeric anthocyanins were quantified in the 3 unprocessed Pinot noir samples and 3 pomace samples. Data are presented as mean ± SD expressed as µg Malvidin-3-glucoside equivalent per mg dry mass (µgEMV/mg DM). Our data indicate differences in the presence of anthocyanins present in the 3 groups obtained from pomace (R1 vs. R2 *p* = 0.99; R1 vs. R3 *p* = 0.02; R2 vs. R3 *p* = 0.01) compared to the groups obtained from the raw sample (E1 vs. E2 *p* = 0.13; E1 vs. E3 *p* = 0.053; E2 vs. E3 *p* = 0.87) (Data not shown). The average total monomeric anthocyanins found in the unprocessed grape is 0.0496 (µgEMV/mg MS), while an average of 0.008 (µgEMV/mg MS) was found in the residue, which is equivalent to 20% of what was found in the unprocessed grape (Figure 1A) (*p* ˂ 0.0001). These results are relevant since an important part of the anthocyanins are transferred to the wine from the skin during vinification process. The percentage seems consistent when the concentrations of total anthocyanins in unprocessed grapes are compared with previous studies. For example, studies carried out on pomace from other grape strains have shown higher concentrations of total monomeric anthocyanins [33] as reports of other strains with lower content have also been seen [42]. On the other hand, if we change the way of expressing the results, that is, change the values of ε and PM of Malvidin-3-glucoside by the values of Cyanidin-3-glucoside in formula 1 (materials and methods), the average of Pinot noir residue is 1.28 mgEC3G/100 g MS, now the data obtained can be compared with other studies with different species. For example, Lillo et al. (2016) [43] spectrophotometrically compared the phenolic compounds of native berries from southern Chile. The anthocyanin concentrations of these berries ranges between 7.6 mg EC3G/100 g DM in *U. molinae* (murtilla) and 3.41 mg EC3G/100 g DM in *A. chilensis* (Maqui), which are equivalent to 5.9 and 2.6 times more concentrations of total monomeric anthocyanins than in Pinot noir pomace. The importance of it lies in that wine companies currently discard pomace or use it as fertilizer for their vineyards. Producing a by-product generated through the extracts of the waste from the wine industry would give it, in addition to a great added value, the possibility of competing strongly with those products generated directly from the production of so-called superfoods. The costs that are not used in the maintenance of the maqui, blueberry, murtilla and calafate, could be deducted from the final value of the product.

Oxidative stress is the process of a free radical generation that includes reactive oxygen species and reactive nitrogen species (ROS and RNS). Increased ROS and RNS concentrations have been linked to the development of various diseases, including cancer and cardiovascular diseases [44,45]. Several methodologies have been conducted to evaluate the antioxidant capacity of natural products [46]. This study evaluates the antioxidant capacity through the Oxygen Radical Absorption Capacity (ORAC). Lately, the ORAC value has been used to standardize the antioxidant activity of herbal and food extracts, widely used as an objective indicator of antioxidant activity in vivo [47]. The ORAC method measures the ability of phytochemicals to neutralize peroxyl radicals [48], since the ORAC antioxidant capacity of the extracts will increase proportionally with their anthocyanin content [49]. In this work, Pinot noir pomace shows an ORAC of 2998 ± 139.2 µmol TE/g d.w. corroborating the association with the concentration of total polyphenols found in this pomace. The results of this research are consistent with the data, showing that the ORAC of the extract generated from the total pomace of the Negramaro strain (2496.75 ± 449.20 µmol TE/g d.w.) is higher than the ORAC generated from the skin extract (1767.85). ± 126.96 µmol TE/g d.w.) [47]. Chitosan-encapsulated grape pomace nanoparticles increased their ORAC from 2058 to 2463 μmol TE/g after digestion [50]. Most polyphenols are degraded along the digestive tract due to pH and digestive enzymes [51]. This encapsulation method allows for an increase in the bioavailability of the polyphenols found in the pomace of the grape without reducing its antioxidant capacity, which in turn allows extrapolating the pharmacological potential of the residue.

Regarding the anthocyanin components of the Pinot noir residue extract (Figure 1B), the predominant component was malvidin-3-glucoside (3.7 ± 2.7 Eq. Malv-3-glu/kg), then peonidin-3-glucoside (0.33 ± 0.24 Malv-3-glu/kg), petunidin-3-glucoside (0.18 ± 0.16 Malv-3-glu/kg), cyanidin-3-glucoside (0.03 Malv-3-glu/kg) and delphinin-3-glucoside (0.05 ± 0.04 Malv-3-glu/kg). These results are consistent with the data obtained in a study that evaluated the anthocyanin components present in the residue of the same strain, finding that malvidin-3-glucoside was the predominant component, while delphinin-3-glucoside was the pigment found in lower concentrations [34,52]. These results are consistent with those of that support the idea that the valuable polyphenolic components present in Pinot noir pomace could be used as phytopharmaceuticals and exert protective properties [34]. Anthocyanins may protect against atherosclerosis and cardiovascular disease through their effects on cellular antioxidant status, oxidative stress, and inflammation [53]. A current study showed that blueberry anthocyanins can improve immune function and reduce metastasis and cancer cell extinction [54]. In addition, an interesting current study showed that the preventive effect of cardiovascular and cerebrovascular diseases of Omega-3 fatty acids can be enhanced when combined with anthocyanins [55]. An investigation studied the effect of blueberry malvidin-3-glucoside on ROS production in HUVEC cells, finding that malvidin-3-galactoside and malvidin-3-glucoside decrease ROS and XO-1 levels and increase SOD levels [16].

### 3.2. In Silico Modelling and Molecular Couplings

The activity of anthocyanins could be associated with the ability to provoke cellular adaptive responses involving the transcription factor Nrf2 by affecting the “nucleophilic tone” of the organism [56]. In this context, the transcription factor Nrf2 has gained relevance due to its ability to inhibit ROS production. Nrf2 in resting conditions binds to Keap1 to be repressed, but once the cell is subjected to oxidative stress, Nrf2 is released from Keap1-mediated repression [57]. Antioxidant genes such as HO-1 and NQO1 are quickly activated to return to homeostasis. A previous publication of our research group showed that Pinot noir pomace can activate Nrf2, which could explain the protective effect of the extract against the cytotoxic effect of polycyclic aromatic hydrocarbons [34].

**Figure 2:**
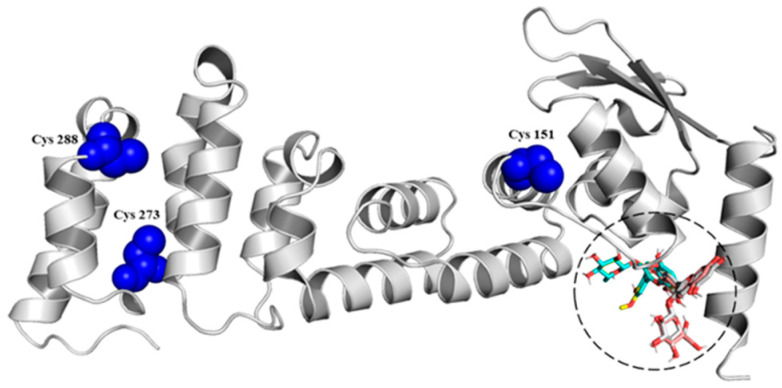
Interaction among the BTB domain of Keap1 protein and different anthocyanins.

Studies have revealed three main cysteine residues in Keap1: Cys^151^ in the BTB domain and Cys^273^ and Cys^288^ in the IVR. These residues are critical for modulating the activity of the E3 ubiquitin ligase of the Keap1-Cul3 complex. Of the three cysteines, Cys^151^ is the main oxidative stress sensor that disrupts the Keap1-Cul3 interaction and causes the dissolution of Nrf2 from Keap1 [58], while Cys^273^ and Cys^288^ are important in changes in the spatial conformation of Keap1 [20]. This research evaluated in silico the interaction of the Cys^151^ and Cys^273−288^ residues of the BTB (Figure 2) and IVR (Data not shown) domains located in the Keap1 protein with the anthocyanins identified in the Pinot noir extract) [34] and quantified in this work. The results showed that anthocyanins have a high affinity for the BTB domain (Table 2). Other studies also showed the potential molecular interaction of some phytochemicals with Cys residues. One study showed that epidermal cells transfected with a mutant Keap1 protein in which Cys^151^ is replaced by serine exhibit a marked reduction in curcumin induced Nrf2 transactivation. Mass spectrophotometric analysis revealed that curcumin binds to Keap1-Cys^151^, supporting that this amino acid is a key target for Keap1 modification, thereby facilitating Nrf2 release [59]. One study found that the pigment betanin can modify cysteine residues, specifically Cys ^151,273,288^, through interaction with their sulfhydryl (SH) groups, allowing the cleavage of the Keap1-Nrf2 interaction [60]. In addition, other antioxidant compounds, such as the flavonoid rutin, can modify Keap1 cysteine residues through oxidation and alkylation, thus activating the Nrf2/ARE pathway [61]. Natural products: Witaferin A [62] and xanthohumol [63] have been seen interacting with cysteine residues in Keap1. Finally, documented molecules that interact with Cys^151^ can be reviewed in Kim and Jeon (2022) [27]. With these results, this research reports that anthocyanins could improve the protection of endothelial cells and prevent consequent cardiovascular diseases due to their dysfunction because of the release of Nrf2 from Keap1, confirming what this working group showed in previous studies [34], yet in vitro studies must be carried out to understand the molecular mechanisms that would allow this protection.

### 3.3. Positive Regulation of HO-1 and NQO1 by HUVEC Pinot Noir Pomace Extract

Once Nrf2 translocates into the nucleus, genes linked to the regulation of oxidative stress are expressed. Among the widely studied genes are those that allow the translation of HO-1 and NQO1 proteins. HO-1 is an enzyme that catabolizes the Heme group, generating equimolar amounts of Biliverdin, ferrous iron, and carbon monoxide [28]. In a general setting, HO-1 modulates the expression of adhesion molecules associated with endothelial cell dysfunction. This effect has been associated with biliverdin production [29]. Similarly, carbon monoxide (CO) has shown to have cytoprotective effects as anti-inflammatory and antioxidant effects. A study by Cheng et al. determined that CO released by carbon monoxide-releasing molecule-2 (CORM-2) inhibits PM-induced inflammatory responses in human oral keratinocytes (HOK) [28]. At low concentrations, CO inhibits vasoconstrictor enzymes such as Endothelin-1 in human pulmonary artery smooth muscle cells [64]. On the other hand, NQO1 is a highly inducible protein in response to multiple forms of stress, including oxidative stress and many protective mechanisms against oxidative damage [31], because its evaluation in this work presents an opportunity to recognize its action against mechanisms linked to the protection of endothelial cells. To evaluate the effect of Pinot noir pomace extract on Nrf2, HO-1 and NQO1 mRNA expression, HUVEC cells were treated with increasing concentrations of the extract (100–400 µg/mL).

**Figure 3:**
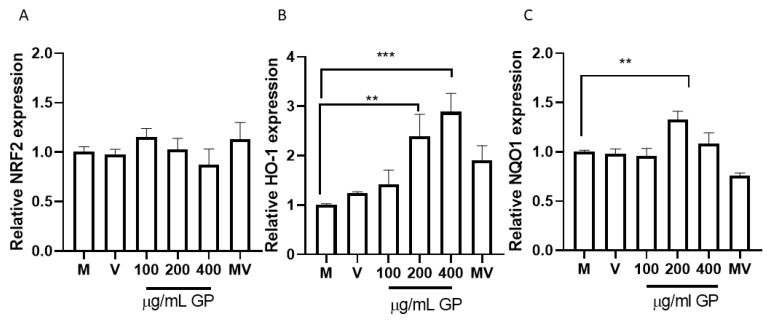
Expression of antioxidant genes, by RT-qPCR, in response to different concentrations of grape pomace (GP). (**A**) Relative NRF2 expression; (**B**) Relative HO-1 expression; (**C**) Relative NQO1 expression. Results are presented as mean ± SEM (*n* = 3). ** *p* ˂ 0.005, *** *p* ˂ 0.0005. M (cells without treatment); V (DMSO ˂ 0.1%) and MV (Malvidin 3-glu). RPL30 was used as normalizer.

As shown in Figure 3A, none extract concentration was able to increase the expression of Nrf2 significantly; however, the concentrations of HO-1 (*p* = 0.0004) and NQO1 (*p* = 0.0084) increased as the extract concentration increased (Figure 3B,C). Malvidin-3-glucoside did not show differences with the control in any performed tests. These results coincide with the in silico analysis (Figure 2) and allow us to understand that, by not increasing the expression of this transcription factor with any of the concentrations evaluated, the anthocyanins present in the pomace do not act on the Nrf2 regulation pathway. These results agree with those obtained by a recent study that evaluated the antioxidant capacity of OxiCyan^®^, a phytocomplex formed by bilberry and spirulina. This study attributed the ROS scavenging activity of OxiCyan^®^ to the anthocyanins found in bilberry, while the gene activation of the ARE/Nrf2 pathway was triggered by spirulina [65].

The results shown are consistent with the results that show the increase in the expression of HO-1 when HUVEC and EA.hy926 cells are treated with different concentrations of witaferin [62]. Note that malvidin is the component found in the highest proportion in the extract (Figure 1B) but does not show a significant effect on the overexpression of HO-1. Then we can infer that the effect shown is the consequence of a synergistic effect more than the effect of each compound separately. In addition, all the anthocyanins quantified in the pomace extract have a high affinity for the BTB binding site of Keap1 (Table 2), which allows to infer more strongly the potential synergistic effect of the components found in pomace extract. A study that used quercetin to evaluate the Nrf2 pathway showed increased expression of NQO1 [66], another molecule linked to cell protection against oxidative stress. An unexpected finding was that NQO1 also showed increased expression when cells were cultured with 200 ug/mL of Pinot noir extract, which may be since the extract also contains significant amounts of this polyphenol [34].

## 4. Conclusions

This research results showed that after wine production, the polyphenolic components present in the skin, pulp and seeds of the berries remain preserved in the Pinot noir residue. The analyzes carried out show the presence of anthocyanins of biological interest, which could provide a high antioxidant capacity. We have shown that the presence of the main glycosylated anthocyanins identified in the extract have an affinity for the BTB domains in Keap1, suggesting a possible interaction of the anthocyanins found in the residue with Cys^151^ and that anthocyanins play a very important role in Nrf2 activation and release, while at the same time not promoting its transcription. These data were validated in vitro, showing that the extract allows the expression of HO-1 and NQO1, genes linked to the regulation of oxidative stress. These preliminary results support the idea that the polyphenolic components extracted from Pinot noir pomace could be used as a source of natural antioxidants to protect endothelial cells from chemical agents that deregulate the oxidative balance. However, more research is required to confirm the molecular mechanisms that could explain the protective effect that Pinot noir pomace extract exerts on endothelial cells.

## Figures and Tables

**Table 1:** Primers sequences used in the analysis of gene expression.

Gene	Accession Number	Primers		Ref.
		Forward	Reverse	
NRF2	NM_001313903	GAATTGCCTGTAAGTCCTGGTC	GGTGAAGGCTTTTGTCATTTTC	[40]
HO-1	NM_002133	CTTCTTCACCTTCCCCAACA	ATTGCCTGGATGTGCTTTTC	[40]
NQO1	NM_001025433	AGACCTTGTGATATTCCAGTTC	GGCAGCGTAAGTGTAAGC	[41]
RPL30	NM_000989	ATGGTGGCCGCAAAGAAGA	TCTGCTTGTACCCCAGGACGTACT	--

NRF2: Nuclear factor erythroid 2 (NF-E2)-related factor 2; HO-1: heme oxygenase-1; NQO1: quinone oxidoreductase 1; RPL30: Ribosomal protein L30.

**Table 2:** Molecular docking results of the anthocyanins evaluated and BTB domain.

Ligand	Binding Affinity (kcal/mol)
Anthocyanins	
Cyanidin-3-glucoside	−6.9
Delphinidin-3-glucoside	−6.5
Malvidin-3-glucoside	−6.6
Peonidin-3-glucoside	−6.6
Petunidin-3-glucoside	−6.5

## Data Availability

Data is contained within the article.
